# *Candida albicans* Hap43 Domains Are Required under Iron Starvation but Not Excess

**DOI:** 10.3389/fmicb.2017.02388

**Published:** 2017-12-01

**Authors:** Volha Skrahina, Matthias Brock, Bernhard Hube, Sascha Brunke

**Affiliations:** ^1^Department of Microbial Pathogenicity Mechanisms, Leibniz Institute for Natural Product Research and Infection Biology - Hans Knoell Institute, Jena, Germany; ^2^Fungal Genetics and Biology Group, School of Life Sciences, University of Nottingham, Nottingham, United Kingdom; ^3^Center for Sepsis Control and Care, Jena University Hospital, Jena, Germany; ^4^Friedrich Schiller University, Jena, Germany

**Keywords:** iron homeostasis, iron deficiency and toxicity, transcriptional regulation, hemoglobin, fungal pathogenicity

## Abstract

Iron availability is a central factor in infections, since iron is a critical micronutrient for all living organisms. The host employs both iron limitation and toxicity strategies to control microbial growth, and successful pathogens are able to tightly coordinate iron homeostasis in response to changing iron levels. As a commensal and opportunistic pathogen, *Candida albicans* copes with both iron deficiency and excess *via* the precise regulation of iron acquisition, consumption and storage. The *C. albicans* transcription factor Hap43 is known to be required for the iron starvation response, while specific domains of its ortholog, HapX, in *Aspergillus fumigatus*, were recently shown to regulate iron uptake and consumptions genes under both low and high iron levels. Therefore, we investigated the contribution of *C. albicans* Hap43 domains in response to changing iron levels. We found the C-terminus of Hap43 to be essential for the activation of iron uptake genes during iron starvation, whereas, in contrast to *A. fumigatus*, Hap43 was not required in mediating adaptation to iron resistance. These data indicate that the generally conserved metal acquisition systems in fungal pathogens can show individual adaptations to the host environment.

## Introduction

Iron is a trace metal with crucial roles in a multitude of biological processes such as oxidative phosphorylation, oxygen transport, and oxidative stress detoxification. Thus, iron availability is recognized as a central factor in infections: the host restricts access to iron in order to prevent microbial growth in a process known as “nutritional immunity” (Weinberg, 1975), while pathogens employ various strategies to obtain the metal from the host (Hood and Skaar, 2012). However, an excess of iron can become toxic due to the production of hydroxyl radicals in the Fenton reaction (Halliwell and Gutteridge, 1984). In addition to iron limitation, the host employs the toxic properties of iron to control infections. Therefore, pathogens have evolved to cope not only with iron limitation, but also with high iron levels (Chen et al., 2011; Gsaller et al., 2014; Xu et al., 2014; VanderWal et al., 2017). In conclusion, a successful pathogen requires mechanisms that tightly regulate iron homeostasis.

As a commensal and opportunistic pathogen, *Candida albicans* is able to cope effectively with both iron limitation and excess. In order to survive within the host during severe iron limitation, *C. albicans* activates its iron acquisition machinery and represses iron consumption pathways (Lan et al., 2004; Chen et al., 2011). Iron acquisition in *C. albicans* encompasses the use of the host's iron transport and storage proteins; acquisition of (xeno-)siderophores (produced by other microorganisms, as *C. albicans* does not synthesize its own); and direct import *via* its high-affinity reductive iron uptake system. In the human body 70% of iron is contained in heme, which is mainly found in hemoglobin (Doherty, 2007), and which *C. albicans* can use as a source of iron (Moors et al., 1992; Santos et al., 2003). *C. albicans* hemoglobin utilization relies on the extracellular heme receptors, Rbt5 and Pga7, as well as the heme oxygenase Hmx1 (Kulkarni et al., 2003; Pendrak et al., 2004; Kuznets et al., 2014). Iron bound to xeno-siderophores is sequestered *via* Sit1, a siderophore-specific membrane importer (Heymann et al., 2002; Hu et al., 2002). The high-affinity reductive uptake system finally consists of reductases, which reduce Fe^3+^ to Fe^2+^; ferroxidases, responsible for re-oxidation to Fe^3+^, and permeases, which import Fe^3+^ into the cell (Ramanan and Wang, 2000; Chen et al., 2011). Under iron excess, *C. albicans* down-regulates iron acquisition systems and up-regulates iron-requiring processes such as biosynthesis of Fe-S clusters, heme, and heme-containing enzymes, respiration, and the tricarboxylic acid (TCA) cycle (Lan et al., 2004). Additionally, under iron excess, Ccc1, a vacuole iron importer, is induced and iron is transported into the vacuole and thereby rendered harmless to the cell (Xu et al., 2014).

*C. albicans* iron homeostasis was shown to be tightly regulated depending on the environment's iron availability. Under high iron levels, Sfu1, a GATA family transcription factor (TF), represses iron acquisition genes and Sef1, a Zn(2)Cys(6) transcription factor. Under low iron levels, Sef1 in turn activates iron uptake genes and Hap43, a part of the CCAAT-binding complex (CBC), which then represses Sfu1 and genes involved in iron-dependent processes (Chen et al., 2011). Hap43 carries out its function in the regulation of gene expression *via* its interaction with the CBC (Baek et al., 2008). The CBC itself is a conserved heterotrimeric DNA-binding complex present in fungi, plants, and mammals (Mantovani, 1999). Hap43 homologs, which mediate the gene regulation after CBC binding to DNA, can however only be found in fungi. In *Aspergillus nidulans* (HapX), *A. fumigatus* (HapX), *Cryptococcus neoformans* (HapX), and *C. albicans* (Hap43), the Hap/CBC complex mediates both positive and negative gene regulation in response to changing iron levels. Consequently, Hap43 is required for virulence in *A. fumigatus, C. neoformans*, and *C. albicans* (Schrettl et al., 2008, 2010; Jung et al., 2010; Hsu et al., 2011; Singh et al., 2011).

**Figure 1:**
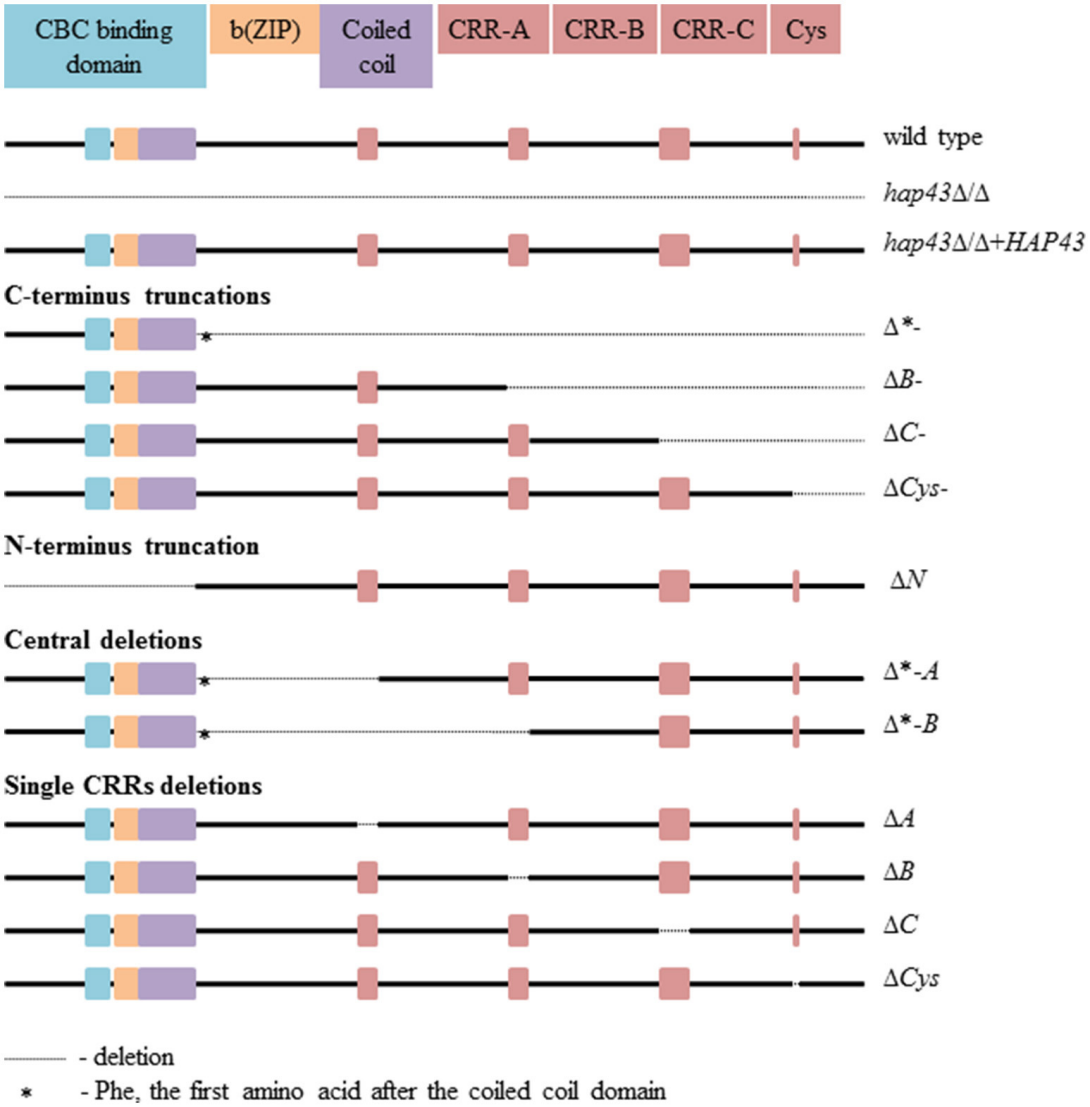
The Hap43 domains organization in the wild type and 13 mutants.

The Hap43 domains found in *C. albicans* are conserved among pathogenic fungi (Gsaller et al., 2014). The N-terminus includes the CCAAT-binding, b(ZIP), and coiled-coil domains, while the C-terminus consists of three cysteine-rich regions (CRR, each with four cysteine (Cys) residues), designated A, B, and C, and a single cysteine (Figure 1). Interestingly, *A. fumigatus* HapX was recently found to be essential under both iron limitation and excess (Gsaller et al., 2014). The HapX C-terminus is essential for growth during iron starvation, *via* the activation of the iron uptake gene (*mirB*) and the repression of iron consuming processes. In contrast, the highly conserved CRR-A, CRR-B, and—to a lesser degree—CRR-C domains of HapX allow growth under iron excess *via* the activation of genes required for vacuolar iron sequestration (*cccA*) and iron consumption by iron-sulfur cluster containing enzymes (*leuA*) and heme biosynthesis (*hemA*) (Gsaller et al., 2014).

*C. albicans* Hap43 is known to be essential during iron starvation (Baek et al., 2008; Chen et al., 2011; Hsu et al., 2011), but in contrast to *A. fumigatus*, the contributions of the individual Hap43 domains have not been investigated. Here, we studied the role of each individual Hap43 domain and found that, similar to *A. fumigatus*, the C-terminus of Hap43 is essential for the activation of iron uptake genes. Since *A. fumigatus* HapX mediates resistance against high iron levels, we investigated the function of *C. albicans* Hap43 under similar conditions. Surprisingly, we found that Hap43 appears to play only a minor role under iron excess in *C. albicans*, in stark contrast to *A. fumigatus*.

## Materials and methods

### Media and growth conditions

*C. albicans* strains were routinely grown in YPD (1% yeast extract, 2% Bacto peptone, 2% glucose). Transformants were selected on SD medium (0.67% yeast nitrogen base [Difco], 2% glucose, 2% Oxoid agar) which was supplemented with the appropriate amino acids and/or uridine. For phenotypic profiling under iron starvation, cells were grown in SD (0.69% yeast nitrogen base without iron [Formedium], 2% glucose). For transcriptional profiling under iron starvation, cells were grown in SD (0.69% yeast nitrogen base without iron [Formedium], 2% glucose, Bathophenanthrolinedisulfonic acid disodium salt (BPS) 0.5 mM [Alfa Aesar]). For phenotypic profiling under high iron levels in liquid media, cells were grown at 30° in SD buffered to pH 4.0 (0.69% yeast nitrogen base without iron [Formedium], 2% glucose, citric acid 59 mM, sodium citrate 41 mM, FeCl_3_ 30 or 2 mM). For phenotypic profiling under high iron levels on solid media, cells were grown at 30° on SD buffered to pH 4.0 (0.69% yeast nitrogen base without iron [Formedium], 2% glucose, 2% Oxoid agar, citric acid 59 mM, sodium citrate 41 mM, FeCl_3_ 5 mM (the highest iron concentration which allowed the medium to solidify). For hemoglobin drop tests, cells were grown at 37° on SD buffered to pH 7.6 (0.69% yeast nitrogen base without iron [Formedium], 2% glucose, 2% Oxoid agar, citric acid 6.35 mM, Na_2_HPO_4_ 187.3 mM, 0.07 mg/ml hemoglobin from bovine blood [Fluka], BPS 0.5 mM [Alfa Aesar]).

### Plasmid and strain construction

The *C. albicans* strains used in this study are listed in Table S2. Plasmids used in this study were constructed using the In-Fusion HD cloning kit [Takara Bio USA] and checked *via* restriction enzyme digestion, PCR, and Sanger sequencing using the primers listed in Table S3. Deletion strains produced in this study were generated in the BWP17 background (Wilson et al., 1999) as previously described (Gola et al., 2003; Walther and Wendland, 2003). All strains were verified by colony PCR. Primers used for mutant production and verification are listed in Table S3. Complementation plasmids were generated by amplifying the gene of interest, including the upstream and downstream intergenic regions followed by cloning into CIp10 (Murad et al., 2000). Resultant complementation constructs were linearized with *Stu*I and used to transform strains as stated above.

### Growth curve analyses

For iron starvation phenotypic profiling, strains were grown in YPD overnight, washed four times in nanopure water and inoculated to OD 0.005 in SD without iron for a first round of starvation to largely deplete internal iron storage. Afterwards, the cells were washed again in nanopure water and inoculated to OD 0.005 in SD without iron for the second starvation phase. During this time, they were placed in the reader [TECAN infinite M200Pro], grown at 30°C, and OD 600 was determined after 30 s of orbital shaking every 30 min. For phenotypic profiling under high levels of iron, the lag phase yeasts were washed only once.

### PCR and quantitative real-time reverse transcription-PCR (qRT-PCR)

To determine gene expression levels, cells were grown in YPD overnight (30°C, 180 rpm) and then washed four times with nanopure water. All tested strains were inoculated to OD 0.2 either in 10 ml iron-free SD with additional BPS 0.5 mM, or in iron-free SD supplemented with FeCl_3_ 20 mM for 4 h. For the shift assay, strains were grown in YPD overnight, washed four times in nanopure water, inoculated to OD 0.2 in iron-free SD with additional 0.5 mM BPS and incubated for 4 h. For the shift, these strains were washed once in nanopure water and inoculated to OD 0.2 in iron-free SD supplemented with FeCl_3_ 50 μM and incubated for 30 min. Then, the yeasts were frozen in liquid nitrogen, followed by a total RNA extraction [Qiagen RNeasy]. The total RNA was treated with DNase [Epicenter Baseline-ZERO] and purified using the kit [Qiagen RNeasy]. RNA quality was verified *via* the bioanalyzer instrument [Agilent]. The RNA concentration was determined using a NanoQuant plate in the reader [TECAN infinite M200Pro]. For each sample, 500 ng RNA was transcribed into cDNA, which was checked *via* PCR for genomic DNA (gDNA) contamination using intron-spanning amplicons (Table S3). In addition, standard PCRs with cDNAs from wild type and mutant strains as template for *HAP43* amplification were performed (Figure S1). Finally, a total amount of 1.85 ng cDNA was used for each qRT-PCR using the fluorescent dye EvaGreen [Bio&Sell] in the thermal cycler [CFX96™ Real-Time System Bio-Rad] in biological and technical triplicates. Expression rates were determined relative to the housekeeping gene *ACT1* and analyzed using the Software [Bio-Rad CFX Manager]. For the shift assay the qRT-PCR was performed in five biological replicates, each with three technical replicates, and normalized to the transcript levels of *ACT1* in each strain and to the wild-type transcript levels. All primers are listed in Table S3. Statistical analyses were performed by one way ANOVA followed by Dunnet tests, where the mean of each mutant strain was compared with the mean of the wild type strain.

## Results

### Mutated *Hap43* variants are transcribed under iron depletion

In order to dissect the individual functions of the different Hap43 domains, we created 13 mutant strains: a full-length *hap43*Δ/Δ deletion and a *hap43*Δ/Δ+*HAP43* complemented strain; C-terminal truncations covering the CRRs (Δ^*^-, Δ*B*-, Δ*C*-, and Δ*Cys*-); an N-terminally truncated strain (Δ*N*); deletions of central parts of Hap43 (Δ^*^-A and Δ^*^-B); and finally targeted deletions of the CRRs only (Δ*A*, Δ*B*, Δ*C*, and Δ*Cys*). The locations of all deletions are shown in Figure 1, and the genetic identity of all strains was checked by Sanger sequencing of the *HAP43* locus. The *HAP43* variants were actively transcribed in all strains, except, of course, for *hap43*Δ/Δ, as evidenced by specific PCR amplicons obtained from cDNAs of all strains grown under low iron levels (Figure S1).

### Both C- and N-terminus, but not the cysteine residues, are essential for Hap43 function under low iron

**Figure 2:**
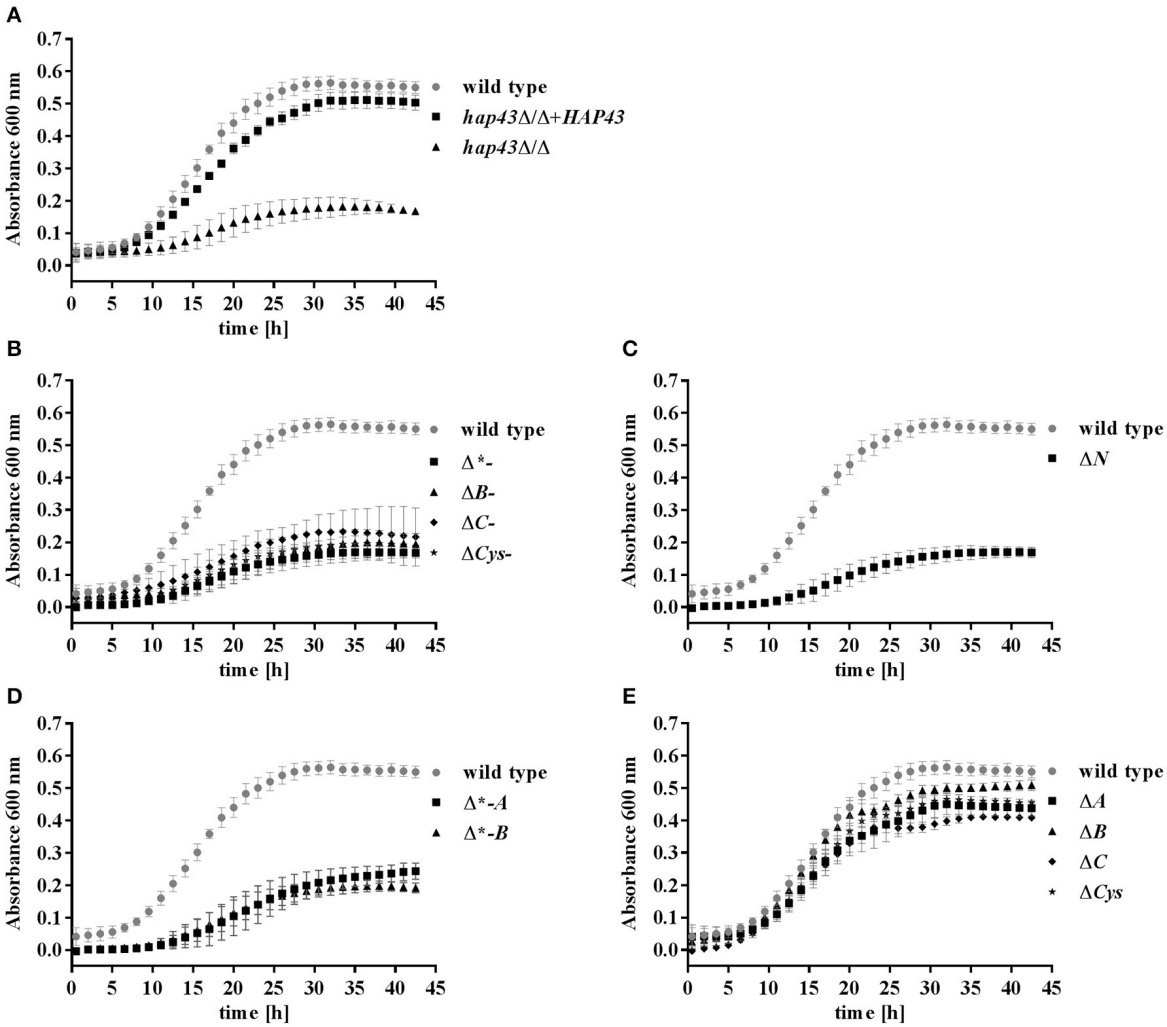
Phenotypic profiling of the wild type and mutant strains under iron limitation. Strains were pre-starved 24 h for iron, and then grown in SD w/o iron with a starting OD of 0.005. **(A)** Wild type, *hap43*Δ/Δ, *hap43*Δ/Δ+*HAP43*; **(B)** C-terminus truncations: Δ^*^-, Δ*B*-, Δ*C*-, and Δ*Cys*-; **(C)** the N-terminus truncation: Δ*N*; **(D)** central deletions: Δ^*^-A and Δ^*^-B; **(E)**; and single CRR deletions: Δ*A*, Δ*B*, Δ*C*, Δ*Cys*.

Having established the expression of all *HAP43* variants, we went on to investigate the growth of wild type and mutant strains in an iron-free medium (SD w/o Fe). A 24 h pre-starvation in the absence of iron was performed in order to deplete the internal iron storage. As expected (Baek et al., 2008), *hap43*Δ/Δ exhibited a growth defect under this iron-depleted condition in comparison to both wild type and *hap43*Δ/Δ+*HAP43* complemented strains (Figure 2A). Mutants truncated at the C-terminus, N-terminus, and deletions of the central regions (to a lesser degree than C- and N-truncations) showed growth defects in SD w/o Fe (Figures 2B–D). In contrast, the mutants lacking the A, B, or C CRRs or the individual Cys retained wild type-like growth during iron depletion (Figure 2E), indicating the dispensability of these regions for regulation of iron uptake.

### Hemoglobin utilization requires both the C- and N-terminus of Hap43

We performed a transcriptional screening of iron-related genes after 8 h iron limitation in both wild type and *hap43*Δ/Δ *via* qRT-PCR. All expression levels were normalized to gene expression levels of the same gene in wild type cells grown in YPD (a medium with sufficient iron). These analyses revealed *RBT5* as one of the most highly up-regulated genes in the wild type upon iron starvation, with a more than 30-fold increase in mRNA abundance. This induction was severely reduced, albeit still present at low levels in the *hap43*Δ/Δ strain (Table S1). Hap43 has previously been shown to positively regulate the expression of *RBT5, PGA7*, and *HMX1*, the key players of the hemoglobin utilization machinery, during iron deprivation (Chen et al., 2011; Singh et al., 2011; Kuznets et al., 2014). In agreement, the expression of *PGA7* and *HMX1* was down-regulated in the *hap43*Δ/Δ strain in comparison to the wild type in our screening. Therefore, we focused in more detail on the regulation of the hemoglobin utilization pathway genes in the wild type and all mutant strains.

**Figure 3:**
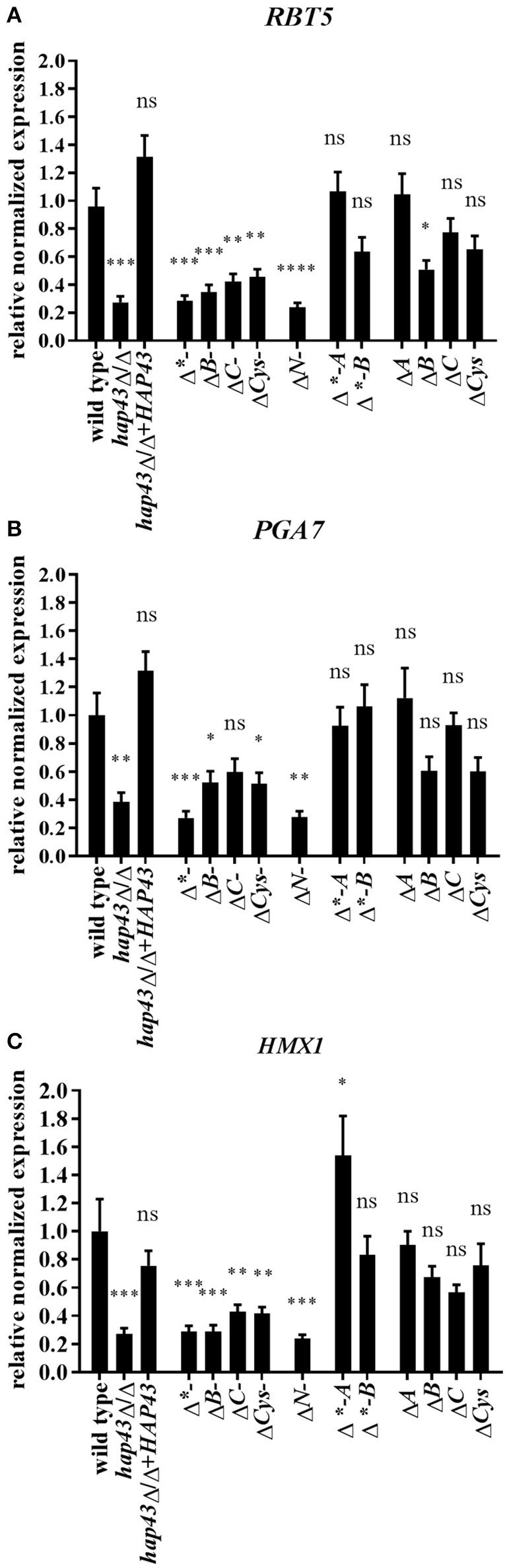
Transcriptional profiling of hemoglobin uptake machinery genes under iron starvation. *RBT5* **(A)**, *PGA7* **(B)**, and *HMX1* **(C)** transcript levels were measured by qRT-PCR in wild type and all mutant strains. The expression was normalized to the transcript levels of *ACT1* in each strain. Asterisks indicate statistical significance compared to the wild type strain (^*^p ≤ 0.05; ^**^p ≤ 0.01; ^***^p ≤ 0.001; ns, *p* > 0.05).

The expression levels of *RBT5, PGA7*, and *HMX1* were measured after 4 h of growth under iron depletion and normalized to the transcript levels of *ACT1* in each strain (Figure 3). Truncation of both the C- or the N-terminus of Hap43 led to a reduced induction of *RBT5, PGA7*, and *HMX1* transcript levels compared to the wild type (Figures 3A–C), in agreement with the growth defect seen under iron depletion for these strains (Figures 2B,C). Surprisingly, deletions of the central regions of Hap43 did not abolish the induction of hemoglobin up-take genes (Figures 3A–C), and *HMX1* transcript levels were even higher in the Δ^*^-A strain, than in the wild type (Figure 3C), although these strains (Δ^*^-A and Δ^*^-B) exhibited similar growth defects under iron-deficient conditions (Figure 2D). Finally, all single CRR deletions did not change the regulation of the hemoglobin up-take machinery genes, with the exception of the Δ*B* strain, where *RBT5* transcription was slightly reduced compared to the wild type (Figure 3A)—mirroring the dispensability of the CRRs in our iron-depleted medium (Figure 2E).

**Figure 4:**
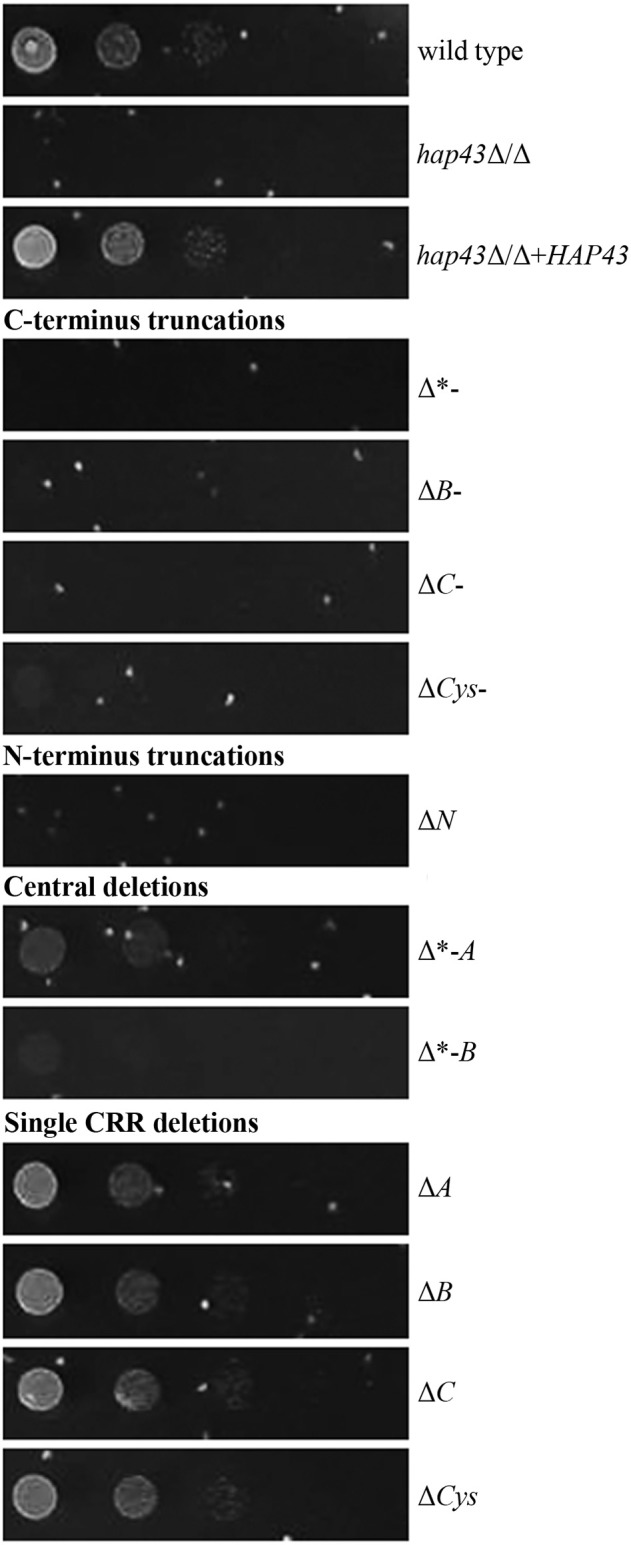
Phenotypic profiling of the wild type and mutant strains in the presence of hemoglobin as the sole iron source. Strains were pre-starved 24 h for iron, and then the drop test was performed at 37° on SD (buffered to pH 7.6 and hemoglobin as the sole source of iron) and on YPD, where all strains retained wild type-like growth (not shown).

To investigate whether the mutants are still able to use hemoglobin as an iron source, all strains were pre-starved for 24 h in the absence of iron and shifted to a medium with hemoglobin as the sole source of iron. C- and N-terminal truncations led to an inability to grow with hemoglobin alone, whereas single CRRs deletions had no effect on growth (Figure 4), in agreement with our transcriptional data. Strains with deletions of central domains of Hap43 showed different phenotypes: the Δ^*^-A strain remained able to use hemoglobin as an iron source, whereas the Δ^*^-B strain displayed a severe growth defect.

### Hap43 is not required for growth or gene regulation under iron excess

**Figure 5:**
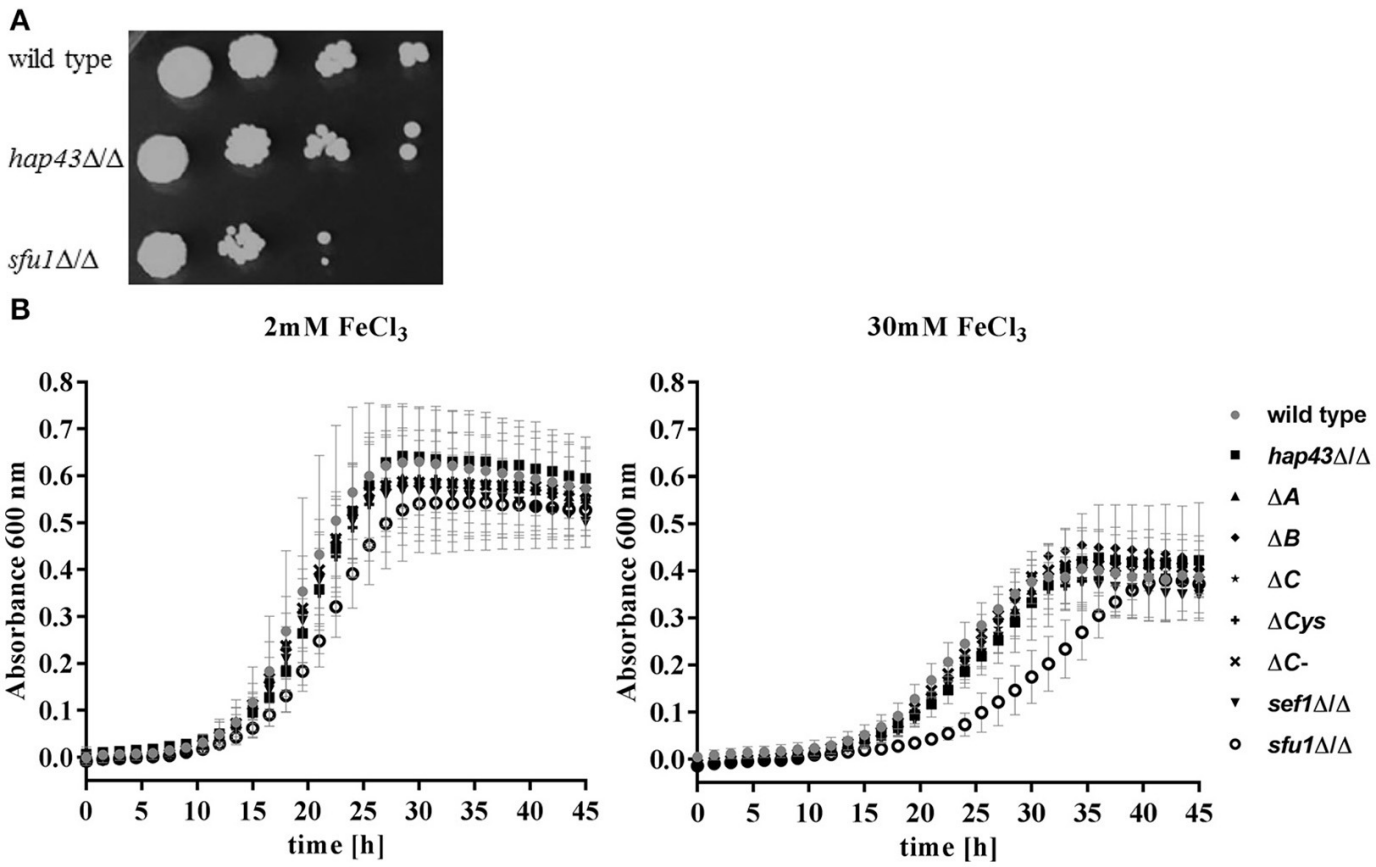
Phenotypic profiling of the wild type and mutant strains under high iron levels. **(A)** Strains were grown on SD plates with 5 mM iron. **(B)** Strains were inoculated to OD 0.005 either in SD with 2 mM iron (iron sufficiency) or in SD with 30 mM iron (iron excess).

In *A. fumigatus*, HapX is essential in both low and excessively high iron environments (Gsaller et al., 2014). However, the functional role of *C. albicans* Hap43 in adaptation to high iron has not yet been investigated in detail. We therefore tested the ability of *hap43*Δ/Δ to grow under iron excess on solid media. The *sfu1*Δ/Δ strain was included as a control, as it was previously shown that Sfu1 represses iron up-take genes in the presence of high levels of iron (Lan et al., 2004; Chen et al., 2011). As expected, *sfu1*Δ/Δ was sensitive to elevated environmental iron. However, *hap43*Δ/Δ grew nearly identically to the wild type on this medium (Figure 5A). We therefore tested growth in liquid medium containing 2 and 30 mM ferric iron (Figure 5B). As expected, the growth of all strains was reduced under iron excess, but we found no difference in the growth of wild type, *hap43*Δ/Δ, CRRs deletion (Δ*A*, Δ*B*, Δ*C*, Δ*Cys*) and the C-terminal truncation (ΔC-) strains. A deletion of *SEF1*, which is known to control *HAP43* expression under iron limitation (Chen et al., 2011) was similarly without visible effect. Only *sfu1*Δ/Δ, which was included as a control for iron excess stress, was defective in growth (Figure 5B). These data suggest that, unlike *A. fumigatus* HapX, *C. albicans* Hap43 or its individual domains are not involved in the response to elevated environmental iron.

**Figure 6:**
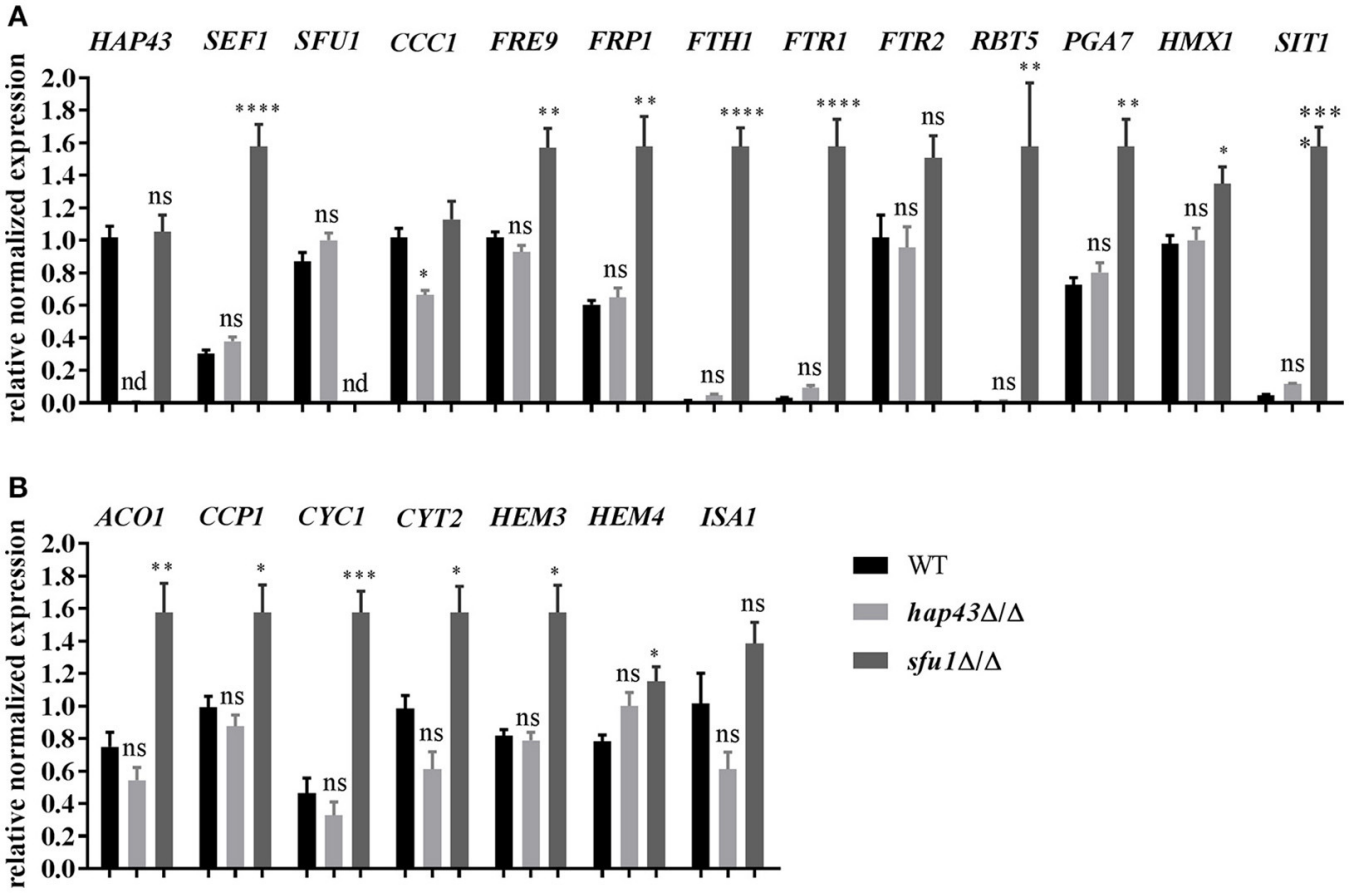
Transcriptional profiling of iron starvation **(A)** and consumption **(B)** genes under iron excess (20 mM FeCl_3_). The expression of iron homeostasis genes was checked *via* qRT-PCR in wild type, *hap43*Δ/Δ, and *sfu1*Δ/Δ strains. The expression was normalized to the transcript levels of *ACT1* in each strain. Asterisks indicate statistical significance compared to the wild type strain (^*^p ≤ 0.05; ^**^p ≤ 0.01; ^***^p ≤ 0.001; ^****^p ≤ 0.0001; ns, *p* > 0.05).

To further investigate this hypothesis, the mRNA levels of several genes involved in iron homeostasis were measured in wild type, *hap43*Δ/Δ, and *sfu1*Δ/Δ cells under conditions of iron excess. Expression analyses included the determination of transcription factor genes (*HAP43, SEF1*, and *SFU1*), genes essential for the reductive iron uptake (*FRE9, FRP1, FTH1, FTR1*, and *FTR2*), hemoglobin utilization (*RBT5, PGA7*, and *HMX1*), siderophore transport (*SIT1*), and vacuolar iron import (*CCC1*) (Xu et al., 2014). Deletion of the *SFU1* repressor gene resulted in an inappropriate overexpression of all iron uptake genes even under iron excess (Figure 6A). These data are in agreement with previous findings (Lan et al., 2004) and may explain the severe growth defect of *sfu1*Δ/Δ under excessive iron (Figure 5). In stark contrast, *hap43*Δ/Δ exhibited an expression profile of iron uptake genes very similar to the wild type, except for *CCC1*, which was significantly less transcribed in *hap43*Δ/Δ in comparison to the wild type. This supports our notion that Hap43 does not play a major role in *C. albicans* transcriptional regulation under conditions of high iron.

Genes with roles in iron utilization are generally transcriptionally induced by *C. albicans* in high-iron environments (Lan et al., 2004). We therefore analyzed transcript levels of iron consuming genes in wild type, *hap43*Δ/Δ, and *sfu1*Δ/Δ under steady-state iron excess. Genes coding for aconitase (*ACO1*), heme biosynthesis (*HEM3* and *HEM4*) and for heme-containing proteins (*CCP1, CYC1*, and *CYT1*) were all significantly up-regulated in *sfu1*Δ/Δ, whilst their expression levels in *hap43*Δ/Δ did not differ from the wild type (Figure 6B). Therefore, in the absence of Sfu1, both iron uptake and iron consumption genes are misregulated, which is in accordance with the severe growth defect of *sfu1*Δ/Δ under high iron growth conditions. However, Hap43 is dispensable for gene regulation under conditions of toxic iron levels (Figure 6), which contrasts the bifunctional role of *A. fumigatus* HapX that is important under conditions of iron starvation and iron saturation.

In *A. fumigatus* the lack of HapX abolishes the transcription of genes coding for iron-requiring processes (*acoA, cycA, leuA, hemA*) especially upon a shift from iron-limited to iron-replete conditions (Gsaller et al., 2014). We therefore investigated the transcript levels of *C. albicans* iron consumption genes (*ACO1, CCC1, CCP1, CYC1, CYT2, HEM3, HEM4*, and *ISA1*) after a shift from a 4 h starvation pre-culture to iron-replete conditions. In contrast to *A. fumigatus*, only two of all genes tested (*ACO1* and *CYC1*) were about 2-fold less transcribed in the *hap43*Δ/Δ mutant (Gsaller et al., 2014) (Figure S2). Expression levels of all other decreased <2-fold or were identical to wildtype transcript levels (Figure S2).

## Discussion

Since both iron starvation and excess are harmful to microbial life, all pathogenic organisms must have developed sophisticated mechanisms to cope with iron fluctuations within the host. Under iron deficiency *C. albicans* relies on the transcription factors (TFs) Sef1 and Hap43 to control the expression of iron uptake and consumption genes. Consequently, these are required for full virulence (Chen et al., 2011; Hsu et al., 2011; Singh et al., 2011). In environments of adequate or elevated iron, another factor, Sfu1, governs the repression of iron uptake genes and thus, is essential for commensal growth in the murine gut, a niche thought to be predominantly iron-replete. On the other hand, the gut niche can rapidly change to become an iron limiting environment through microbial competition and by food intake, and consequently Sef1 was also shown to be important for *C. albicans* commensal growth, although to a lesser degree than Sfu1 (Chen et al., 2011). In contrast to *C. albicans*, the filamentous fungi *A. fumigatus, A. nidulans*, and *F. oxysporum*, which do not preferentially colonize human mucosal surfaces, lack Sef1, and iron homeostasis is thought to be maintained by two other TFs. One of these TF is HapX, the Hap43 homolog, which controls gene expression under both low and high iron levels and therefore is essential for virulence (Schrettl et al., 2008, 2010; López-Berges et al., 2012; Gsaller et al., 2014). The second factor is SreA, a Sfu1 homolog, which additionally represses iron uptake genes under high iron levels (Haas et al., 1999; Schrettl et al., 2008). In-depth analysis of *A. fumigatus* HapX domains, which are conserved among the fungi, have shed light on how a single TF is able to both repress and activate genes depending on the iron content of the surrounding environment (Gsaller et al., 2014). Here, we dissected the role of *C. albicans* Hap43 domains in gene regulation in response to different iron levels. To this end, we deleted various regions within the *HAP43* gene and tested the function of the mutated proteins.

Mutants with both C- and N-terminal truncations exhibited growth defects under iron deficiency and were not able to induce the hemoglobin uptake machinery. The HapX C-terminus in *A. fumigatus* is also essential for the iron limitation response (Gsaller et al., 2014), indicating the conserved function of the C-terminus in both species. This functional similarity is further supported by the 41% amino acid sequence identity between the Hap43 and HapX C termini (Gsaller et al., 2014). The N-terminus of Hap43 is known to be necessary for the Hap/CBC complex assembly, which is required for regulation of gene expression under iron deficiency (McNabb and Pinto, 2005; Hortschansky et al., 2007; Baek et al., 2008; Hsu et al., 2013). Overall, despite the deletion of Hap43 central parts, the transcription factor remained functional, which indicates that the central part of Hap43 is not strictly required for gene regulation. However, as a minor defect was observed, the central Hap43 region might be required for proper protein folding and stability. Like in *A. fumigatus*, all single CRR deletions of Hap43 in *C. albicans* were indistinguishable from the wild type under iron deficiency conditions (Gsaller et al., 2014). Therefore, we conclude that only C- and N-termini of Hap43 are crucial for its function under iron deprivation.

As HapX was found to be important in mediating *A. fumigatus* resistance to high iron (Gsaller et al., 2014), it seemed reasonable to assume a similar function for resistance against iron toxicity in *C. albicans*. However, neither complete deletion of *HAP43* nor deletion of individual or all CRRs or a C-terminal truncation changed the growth in media with excess iron. Therefore, we conclude that Hap43 of *C. albicans*, in contrast to *A. fumigatus, A. nidulans*, or *Fusarium oxysporum* (Gsaller et al., 2014), is not involved in the adaptation to iron excess. Interestingly, under iron excess, *CCC1*, the vacuolar iron importer, was significantly less transcribed in *hap43*Δ/Δ compared to the *C. albicans* wild type (Figure 6A). Similarly to these observations in *C. albicans*, HapX in *A. fumigatus* is known to activate the transcription of *cccA*, a *CCC1* homolog, under high iron levels. However, the overexpression of *cccA* alone in *hapX*Δ largely, but not fully, restored the wild type phenotype under iron excess (Gsaller et al., 2014), showing that for full protection from iron excess, HapX must regulate additional processes, like iron acquisition and consumption, which are not regulated by Hap43 in *C. albicans*. Although Hap43 may regulate transcription of *ACO1* and *CYC1* during the shift from iron starved to replete conditions, the decrease in transcript levels due to Hap43 deletion was only minor compared to *A. fumigatus*. For the *hap43*Δ/Δ mutant we only observed a roughly 2-fold reduction, which is very low compared to the data shown for *A. fumigatus* (Gsaller et al., 2014), and limited to few genes even under the immediate up-shift. The difference in the expression of *C. albicans ACO1* and *CYC1* could possibly be better explained by a less severe iron shock for the *hap43*Δ/Δ mutant: Under iron limitation the wild type, but not the *hap43*Δ/Δ mutant, activates iron uptake and represses iron utilization genes (Chen et al., 2011). Upon the shift to 50 μM FeCl_3_, both the active iron import and the repression of iron consumption likely induced a more severe short-term iron shock in the wild type than in the *hap43*Δ/Δ mutant.

Like *A. fumigatus, C. albicans* has another high-iron responsive factor at its disposal, Sfu1 (SreA in *Aspergillus*), which in *C. albicans* seems solely responsible to provide resistance to iron excess (Lan et al., 2004): deletion of *SFU1* resulted in an uncontrolled high expression of genes of the iron uptake machinery under iron replete conditions. Interestingly, genes for iron consuming processes were also upregulated in the *sfu1*Δ/Δ strain, which can probably be best explained as a secondary effect due to excessive influx of iron in this mutant—although even this reaction evidently did not suffice to fully detoxify the iron excess and allow for normal growth. So why are the functions of HapX and Hap43 different? While our set of deletion mutants did not reveal an immediate explanation, *in silico* analyses revealed that Hap43 and Sfu1 of *C. albicans* each contain four CRRs or single cystein, whereas in *A. fumigatus, A. nidulans*, and *F. oxysporum* the HapX homologs contain five CRRs and the Sfu1 homologs—three CRRs. It is tempting to speculate that these differences, *via* iron binding or protein interactions, could explain the crucial role of Sfu1, but not of Hap43, in mediating iron resistance response in *C. albicans*.

Pathogenic fungi seem to have evolved individual variants of common themes in iron homeostasis. For example, *C. albicans* has integrated an additional factor for iron acquisition, Sef1, into the established reciprocally acting pair of GATA factor (Sfu1/SreA) and CCAAT binding complex (with Hap43/HapX) (Chen et al., 2011); *C. glabrata* has combined elements of this system with the vastly different iron regulatory network of *S. cerevisiae* (Gerwien et al., 2016); and *Aspergillus* spp. and related species employ the CCAAT binding complex to counteract both iron starvation and excess. Nevertheless, *A. fumigatus* contains the SreA system, which directly interacts with HapX. Supporting other data, we have shown here that *C. albicans*, high and low iron regulation is not interwoven by Hap43 and Sfu1, but rather form a reciprocal network (Chen et al., 2011) and there is a clear discrimination between iron starvation response (Hap43) and iron excess response (Suf1). This may make sense if we look at the environments these fungi generally face: gradually shifting environments may favor bi-functional receptors for iron starvation and excess in saprophytic pathogens like *A. fumigatus*. In contrast, the gut commensal *C. albicans* likely faces rapidly changing environments, which require a decisive, yes/no type of transcriptional response without overlapping functionality of either receptor. The iron response, thus, reflects the adaptation strategies of these fungi as formed by their environments.

## Author contributions

VS, SB, and BH: designed the sturdy; MB: suggested an additional experiment; VS: performed the experiments; VS and SB: evaluated and interpreted the results; VS, SB, MB, and BH: wrote and revised the manuscript.

### Conflict of interest statement

The authors declare that the research was conducted in the absence of any commercial or financial relationships that could be construed as a potential conflict of interest.

## Supplementary material

Figure S1 PCR amplification of *HAP43* from cDNA. 1, 16, 17, 28 - 1 kb DNA ladder; 2 - wild type; 3 - *hap43*Δ/Δ; 4 - *hap43*Δ/Δ+*HAP43*; 5 - Δ*B*-; 6 - Δ*C*-; 7 - Δ*Cys*-; 8 - Δ^*^- ; 9 - Δ*N*; 10 - Δ^*^-*A*; 11 - Δ^*^-*B*; 12 - Δ*A*; 13 - Δ*B*; 14 - Δ*C*; 15 - Δ*Cys*; 18, 20, 22, 24, 26 - wild type gDNA; 19, 21, 23, 25, 27 - H_2_O.

Figure S2 Transcriptional profiling of iron consumption genes under shift from iron-limited to iron-replete conditions. The expression of iron homeostasis genes was determined *via* qRT-PCR in wild type, *hap43*Δ/Δ, Δ*A*, Δ*B*, Δ*C*, Δ*Cys*, and Δ*C*-strains. The expression was normalized to the transcript levels of *ACT1* in each strain and to the wild-type transcript levels. Asterisks indicate statistical significance compared to the wild-type strain (^*^*p* ≤ 0.05; ^**^*p* ≤ 0.01).
