# Supplementary figures and images for: Candida albicans Hap43 Domains Are Required under Iron Starvation but Not Excess
Source: Front Microbiol. 2017 Dec 1;8:2388. doi: 10.3389/fmicb.2017.02388 (PMC5717023; doi:10.3389/fmicb.2017.02388)

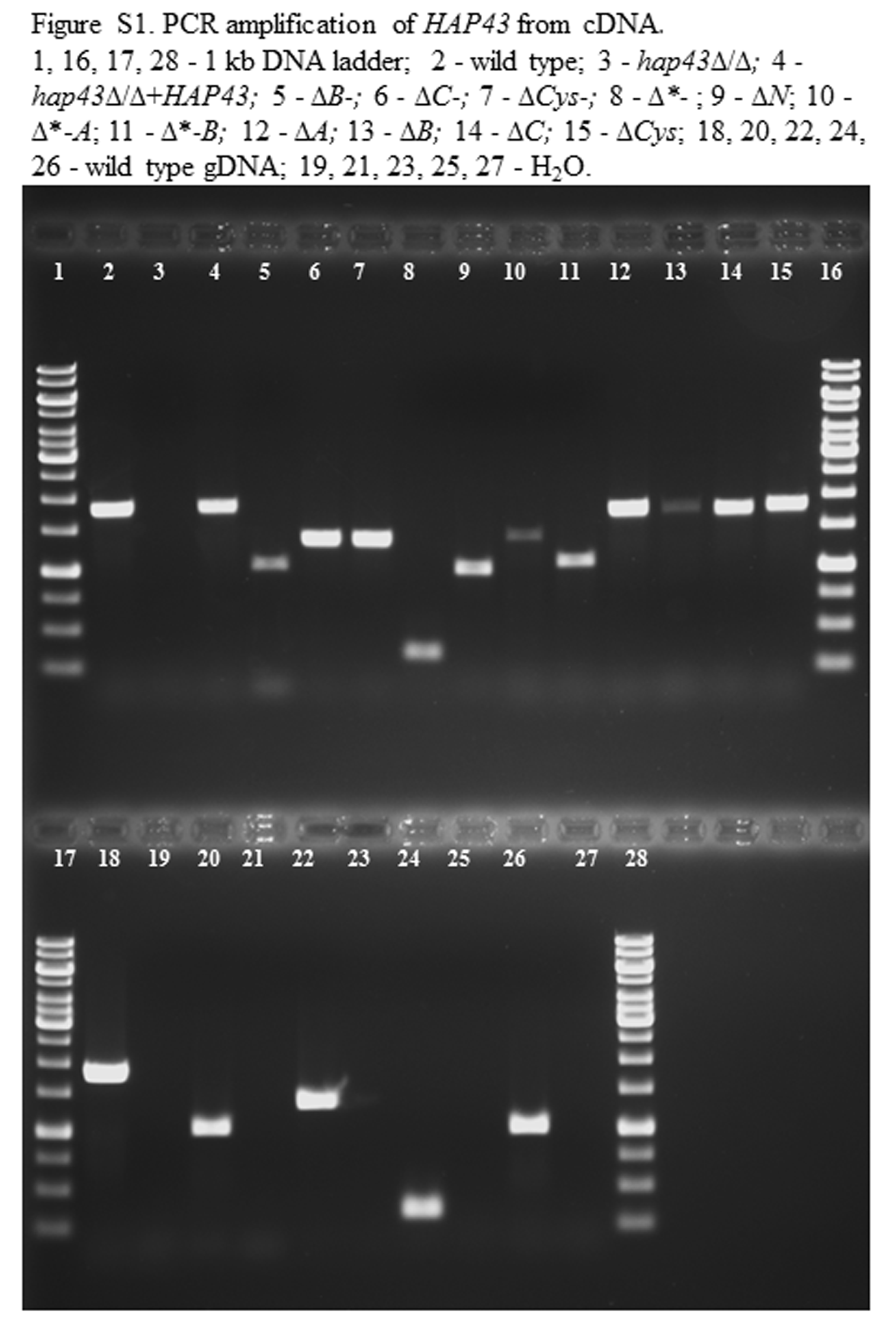

Supplement: Figure S1 — PCR amplification of HAP43 from cDNA. 1, 16, 17, 28 - 1 kb DNA ladder; 2 - wild type; 3 - hap43Δ/Δ; 4 - hap43Δ/Δ+HAP43; 5 - ΔB-; 6 - ΔC-; 7 - ΔCys-; 8 - Δ*- ; 9 - ΔN; 10 - Δ*-A; 11 - Δ*-B; 12 - ΔA; 13 - ΔB; 14 - ΔC; 15 - ΔCys; 18, 20, 22, 24, 26 - wild type gDNA; 19, 21, 23, 25, 27 - H2O. [file Image1.TIF]

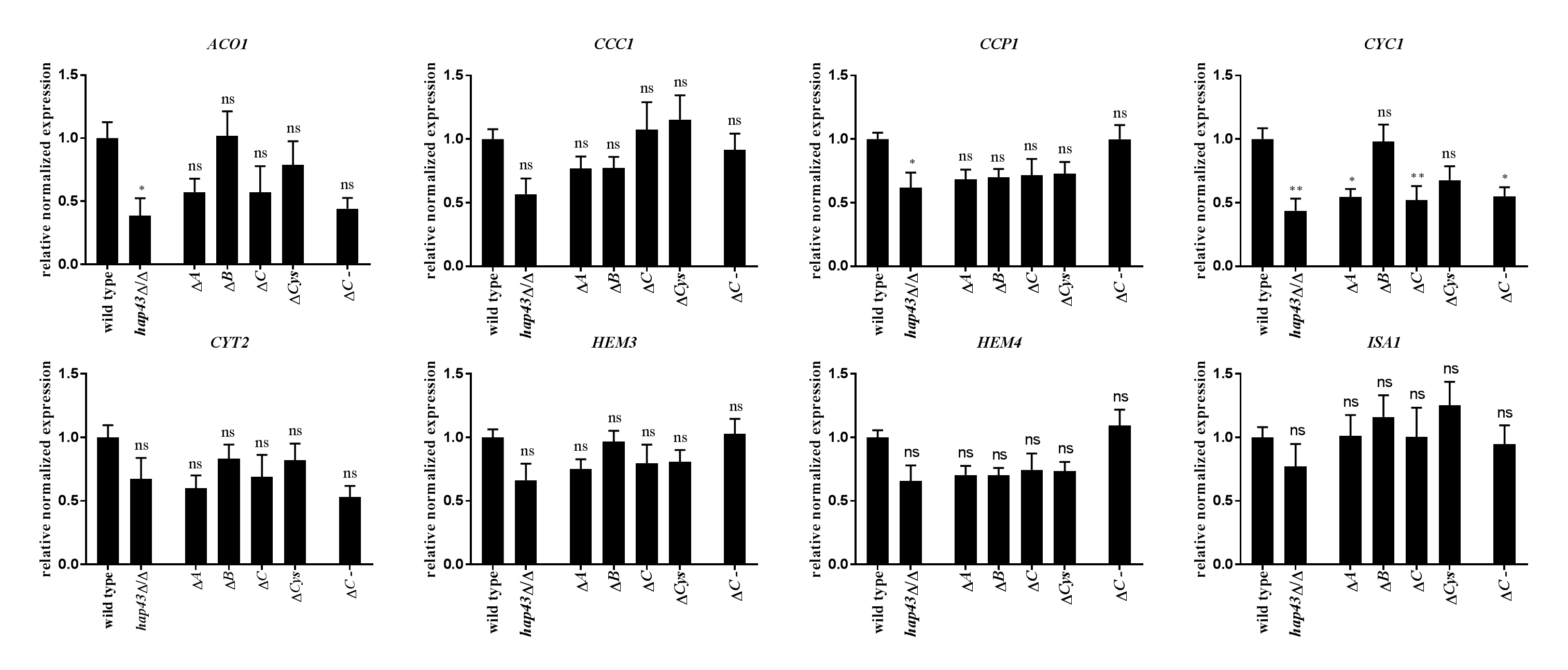

Supplement: Figure S2 — Transcriptional profiling of iron consumption genes under shift from iron-limited to iron-replete conditions. The expression of iron homeostasis genes was determined via qRT-PCR in wild type, hap43Δ/Δ, ΔA, ΔB, ΔC, ΔCys, and ΔC-strains. The expression was normalized to the transcript levels of ACT1 in each strain and to the wild-type transcript levels. Asterisks indicate statistical significance compared to the wild-type strain (*p ≤ 0.05; **p ≤ 0.01). [file Image2.TIF]
