# Supplementary material for: Candida albicans Hap43 Domains Are Required under Iron Starvation but Not Excess
Source: Front Microbiol. 2017 Dec 1;8:2388. doi: 10.3389/fmicb.2017.02388 (PMC5717023; doi:10.3389/fmicb.2017.02388)
Supplement: Supplementary file 3 [file Table1.DOCX]

Supplementary Material

*Candida albicans* Hap43 domains in adaptation to changing iron levels

**Volha Skrahina^1^, Matthias Brock^4^, Bernhard Hube^1,2,3^, Sascha Brunke^1^***

**Supplementary Table S1**. **Transcriptional screening of iron-related genes.** qRT-PCR were performed after 8 h iron limitation in wild type and *hap43*∆/∆. All expression levels were normalized to levels of the wild type grown in YPD.

| relative normalized expression 8 h LIM | | | |  |
| --- | --- | --- | --- | --- |
| gene | wild type | SEM | *hap43*∆/∆ | SEM |
| *ACO1* | 0,17982 | 0,02483 | 6,84661 | 1,93165 |
| *AQY1* | 11,34019 | 1,70029 | 4,26883 | 1,39187 |
| *CCC1* | 0,24524 | 0,02686 | 1,84272 | 0,63177 |
| *CCP1* | 0,27232 | 0,03332 | 1,22665 | 0,30138 |
| *COX5* | 0,08038 | 0,01019 | 0,47787 | 0,18251 |
| *CRD2* | 0,03795 | 0,00579 | 0,22128 | 0,05295 |
| *CYC1* | 0,05906 | 0,00622 | 1,78412 | 0,45564 |
| *CYC3* | 0,19190 | 0,03236 | 5,73029 | 2,86538 |
| *CYT1* | 0,04298 | 0,00624 | 0,31814 | 0,16314 |
| *CYT2* | 0,27211 | 0,03853 | 2,34542 | 1,36418 |
| *FET3* | 1,02114 | 0,11303 | 1,73495 | 0,25015 |
| *FRE10* | 2,49657 | 0,30165 | 13,82030 | 5,35959 |
| *FRE9* | 1,03998 | 0,12083 | 1,92029 | 0,32599 |
| *FRP2* | 1,58378 | 0,23276 | 0,18045 | 0,04791 |
| *FTH1* | 7,21949 | 0,84040 | 7,49049 | 2,69045 |
| *FTH2* | 0,87661 | 0,09850 | 1,64671 | 0,24236 |
| *FTR1* | 5,52018 | 0,62207 | 9,55583 | 2,66719 |
| *FTR2* | 0,98994 | 0,10285 | 1,95934 | 0,29143 |
| *HAP43* | 10,17805 | 1,11220 | 0,02842 | 0,00411 |
| *HEM14* | 0,03026 | 0,00420 | 0,24671 | 0,18373 |
| *HEM3* | 0,70313 | 0,07215 | 2,62374 | 0,60443 |
| *HEM4* | 0,15466 | 0,03125 | 0,79326 | 0,32760 |
| ***HMX1*** | **3,39755** | **0,38568** | **1,47290** | **0,68303** |
| *ISA1* | 0,33787 | 0,03953 | 1,79983 | 1,56821 |
| *MMT2* | 0,96385 | 0,10019 | 1,04663 | 0,55070 |
| *MRS4* | 2,46817 | 0,29723 | 2,19300 | 1,05555 |
| *PGA10* | 1,24220 | 0,16441 | 1,20808 | 1,04092 |
| *PGA26* | 0,14762 | 0,01651 | 0,32651 | 0,05354 |
| ***PGA7*** | **15,69319** | **2,11215** | **13,05899** | **4,34464** |
| ***RBT5*** | **32,33166** | **3,41346** | **13,60853** | **5,26710** |
| *RBT51* | 0,84284 | 0,12646 | 0,75405 | 0,12754 |
| *RIM101* | 1,03458 | 0,12810 | 0,40241 | 0,24433 |
| *SEF1* | 1,17820 | 1,84794 | 1,49410 | 2,09941 |
| *SFU1* | 0,22548 | 0,02432 | 1,05931 | 0,57496 |
| *SIT1* | 7,99422 | 0,83191 | 4,10533 | 1,32237 |
| *SMF3* | 0,85220 | 0,09985 | 0,73210 | 0,14466 |
| *SOD5* | 0,25863 | 0,03908 | 0,04358 | 0,01929 |
| *YFH1* | 0,23650 | 0,02474 | 0,71819 | 0,26429 |
