# Supplementary material for: Candida albicans Hap43 Domains Are Required under Iron Starvation but Not Excess
Source: Front Microbiol. 2017 Dec 1;8:2388. doi: 10.3389/fmicb.2017.02388 (PMC5717023; doi:10.3389/fmicb.2017.02388)
Supplement: Supplementary file 4 [file Table2.DOCX]

Supplementary Material

*Candida albicans* Hap43 domains in adaptation to changing iron levels

**Volha Skrahina^1^, Matthias Brock^4^, Bernhard Hube^1,2,3^, Sascha Brunke^1^***

# Supplementary Table S2. The list of *C. albicans* strains used in this study.

| Strain | Name | Name | Genotype | Reference |
| --- | --- | --- | --- | --- |
| M1477 | BWP17 +CIp30 | wild type | ura3:: imm434/ura3:: imm434 his1::hisG/his1::hisG + CIp30 | ([Citiulo, Jacobsen et al. 2012](#_ENREF_1)) |
| M2517 | *hap43*∆/∆ | *hap43*∆/∆ | ura3:: imm434/ura3:: imm434 his1::hisG/his1::hisG *hap43*::*HIS1*/*hap43*::*ARG4* +CIp10 | this study |
| M2518 | *hap43*∆/∆+*HAP43* | *hap43*∆/∆+*HAP43* | ura3:: imm434/ura3:: imm434 his1::hisG/his1::hisG *hap43*::*HIS1*/*hap43*::*ARG4* +CIp10-*HAP43* | this study |
| M2531 | *hap43*∆/∆+*HAP43*_*^141^a* | ∆**-* | ura3:: imm434/ura3:: imm434 his1::hisG/his1::hisG *hap43*::*HIS1*/*hap43*::*ARG4* +CIp10-*HAP43*_*^141^* | this study |
| M2521 | *hap43*∆/∆+*HAP43*_*^373^c* | ∆*B-* | ura3:: imm434/ura3:: imm434 his1::hisG/his1::hisG *hap43*::*HIS1*/*hap43*::*ARG4* +CIp10-*HAP43*_*^373^* | this study |
| M2523 | *hap43*∆/∆+*HAP43*_*^481^a* | ∆*C-* | ura3:: imm434/ura3:: imm434 his1::hisG/his1::hisG *hap43*::*HIS1*/*hap43*::*ARG4* +CIp10-*HAP43*_*^481^* | this study |
| M2528 | *hap43*∆/∆+*HAP43*_*^583^b* | ∆*Cys-* | ura3:: imm434/ura3:: imm434 his1::hisG/his1::hisG *hap43*::*HIS1*/*hap43*::*ARG4* +CIp10-*HAP43*_*^583^* | this study |
| M2533 | *hap43*∆/∆+*HAP43*_*^142-634^a* | ∆*N* | ura3:: imm434/ura3:: imm434 his1::hisG/his1::hisG *hap43*::*HIS1*/*hap43*::*ARG4* +CIp10-*HAP43*_*^142-634^* | this study |
| M2535 | *hap43*∆/∆+*HAP43*_*^141,281-634^a* | ∆**-A* | ura3:: imm434/ura3:: imm434 his1::hisG/his1::hisG *hap43*::*HIS1*/*hap43*::*ARG4* +CIp10-*HAP43*_*^141,281-634^* | this study |
| M2537 | *hap43*∆/∆+*HAP43*_*^141,386-634^a* | ∆**-B* | ura3:: imm434/ura3:: imm434 his1::hisG/his1::hisG *hap43*::*HIS1*/*hap43*::*ARG4* +CIp10-*HAP43*_*^141,386-634^* | this study |
| M2539 | *hap43*∆/∆+*HAP43*_*^269,281-634^* | ∆*A* | ura3:: imm434/ura3:: imm434 his1::hisG/his1::hisG *hap43*::*HIS1*/*hap43*::*ARG4* +CIp10-*HAP43*_*^269,281-634^* | this study |
| M2540 | *hap43*∆/∆+*HAP43*_*^373,386-634^* | ∆*B* | ura3:: imm434/ura3:: imm434 his1::hisG/his1::hisG *hap43*::*HIS1*/*hap43*::*ARG4* +CIp10-*HAP43*_*^373,386-634^* | this study |
| M2541 | *hap43*∆/∆+*HAP43*_*^481,503-634^* | ∆*C* | ura3:: imm434/ura3:: imm434 his1::hisG/his1::hisG *hap43*::*HIS1*/*hap43*::*ARG4* +CIp10-*HAP43*_*^481,503-634^* | this study |
| M2542 | *hap43*∆/∆+*HAP43*_*^583,585-634^* | ∆*Cys* | ura3:: imm434/ura3:: imm434 his1::hisG/his1::hisG *hap43*::*HIS1*/*hap43*::*ARG4* +CIp10-*HAP43*_*^583,585-634^* | this study |

Citiulo, F., I. D. Jacobsen, et al. (2012). "*Candida albicans* scavenges host zinc via Pra1 during endothelial invasion." PLoS Pathog **8**(6): e1002777.
