# Supplementary material for: Candida albicans Hap43 Domains Are Required under Iron Starvation but Not Excess
Source: Front Microbiol. 2017 Dec 1;8:2388. doi: 10.3389/fmicb.2017.02388 (PMC5717023; doi:10.3389/fmicb.2017.02388)
Supplement: Supplementary file 5 [file Table3.DOCX]

Supplementary Material

*Candida albicans* Hap43 domains in adaptation to changing iron levels

**Volha Skrahina^1^, Matthias Brock^4^, Bernhard Hube^1,2,3^, Sascha Brunke^1^***

# Supplementary Table S3. The list of primers used in this study.

| Name | Sequence 5->3’ | Purpose |
| --- | --- | --- |
| FPUHap43ov  RPDHap43ov | TCGATACCGTCGACCTACATACAGCTAAAAGTGTGTCGTAGTTTATTGG  GGGAACAAAAGCTGGGTTTGTTTGTTTGTTTGTTTGAGATTTTTATAGTAA | Plasmid generation CIp10-HAP43 |
| FPUHap43ov  RPendCoil  FPStopafterHAP43  RPDHap43ov | TCGATACCGTCGACCTACATACAGCTAAAAGTGTGTCGTAGTTTATTGG  CTGTAAGTCAAACTA CTGGTTCTCCACCTTTAATTTATTTATAGTGTT  AAGGTGGAGAACCAG TAGTTTGACTTACAGTATGAAGTATTTCCGACAC  GGGAACAAAAGCTGGGTTTGTTTGTTTGTTTGTTTGAGATTTTTATAGTAA | Plasmid generation  CIp10-*HAP43_^141^* |
| FPUHap43ov  IRPCys  IFPCys  RPDHap43ov | TCGATACCGTCGACCTACATACAGCTAAAAGTGTGTCGTAGTTTATTGG  CTGTAAGTCAAACTA ATTGTCAACTGGAGAATCCTCATTAAAAG  TCTCCAGTTGACAAT TAGTTTGACTTACAGTATGAAGTATTTCCGACAC  GGGAACAAAAGCTGGGTTTGTTTGTTTGTTTGTTTGAGATTTTTATAGTAA | Plasmid generation  CIp10-*HAP43_^373^* |
| FPUHap43ov  I_IIRPCys  I_IIFPCys  RPDHap43ov | TCGATACCGTCGACCTACATACAGCTAAAAGTGTGTCGTAGTTTATTGG  CTGTAAGTCAAACTAGCCACCATCAGAAGAAGTATCCTTG  TCTTCTGATGGTGGCTAGTTTGACTTACAGTATGAAGTATTTCCGACAC  GGGAACAAAAGCTGGGTTTGTTTGTTTGTTTGTTTGAGATTTTTATAGTAA | Plasmid generation  CIp10-*HAP43_^481^* |
| FPUHap43ov  I_II_IIIPRCys  I_II_IIIFPCys  RPDHap43ov | TCGATACCGTCGACCTACATACAGCTAAAAGTGTGTCGTAGTTTATTGG  CTGTAAGTCAAACTATGGAATGAAAATACCACTATTGCTCAG  GGTATTTTCATTCCATAGTTTGACTTACAGTATGAAGTATTTCCGACAC  GGGAACAAAAGCTGGGTTTGTTTGTTTGTTTGTTTGAGATTTTTATAGTAA | Plasmid generation  CIp10-*HAP43_^583^* |
| FPUHap43ov  RPendRromStartCod  FPrAcoiledcoilReg  RPDHap43ov | TCGATACCGTCGACCTACATACAGCTAAAAGTGTGTCGTAGTTTATTGG  GTTTTTAACCAAAAACATGTTGTTCAAATTGAAATTCTAATTATTAT  AATTTGAACAACATGTTTTTGGTTAAAAACTTGGAACAGTTAAAAGGT  GGGAACAAAAGCTGGGTTTGTTTGTTTGTTTGTTTGAGATTTTTATAGTAA | Plasmid generation  CIp10-*HAP43_^142-634^* |
| FPUHap43ov  RPendCII_III_IV  AfterCysI  RPDHap43ov | tcgataccgtcgaccTACATACAGCTAAAAGTGTGTCGTAGTTTATTGG  TAACCCCACAGATTCCTGGTTCTCCACCTTTAATTTATTTATAGTGTT  AAGGTGGAGAACCAGGAATCTGTGGGGTTAAAGGAGCCCAC  gggaacaaaagctggGTTTGTTTGTTTGTTTGTTTGAGATTTTTATAGTAA | Plasmid generation  CIp10-*HAP43_^141,281-634^* |
| FPUHap43ov  RPendCIII_IV  AftCysII  RPDHap43ov | TCGATACCGTCGACCTACATACAGCTAAAAGTGTGTCGTAGTTTATTGG  TTTAGCAGCTTCACGCTGGTTCTCCACCTTTAATTTATTTATAGTGTT  AAGGTGGAGAACCAGCGTGAAGCTGCTAAAGAAGCAGCAAA  GGGAACAAAAGCTGGGTTTGTTTGTTTGTTTGTTTGAGATTTTTATAGTAA | Plasmid generation  CIp10-*HAP43_^141,386-634^* |
| FPUHap43ov  RPBCysIov  FPACysIov  RPDHap43ov | TCGATACCGTCGACCTACATACAGCTAAAAGTGTGTCGTAGTTTATTGG  TAACCCCACAGATTCATCAAAGTTAGCTACATCAGCGGCG  GTAGCTAACTTTGATGAATCTGTGGGGTTAAAGGAGCCC  GGGAACAAAAGCTGGGTTTGTTTGTTTGTTTGTTTGAGATTTTTATAGTAA | Plasmid generation  CIp10-*HAP43_^269,281-634^* |
| FPUHap43ov  RPBCysIIov  FPACysIIov  RPDHap43ov | TCGATACCGTCGACCTACATACAGCTAAAAGTGTGTCGTAGTTTATTGG  TTTAGCAGCTTCACGATTGTCAACTGGAGAATCCTCATTAAAAG  TCTCCAGTTGACAATCGTGAAGCTGCTAAAGAAGCAGC  GGGAACAAAAGCTGGGTTTGTTTGTTTGTTTGTTTGAGATTTTTATAGTAA | Plasmid generation  CIp10-*HAP43_^373,386-634^* |
| FPUHap43ov  RPBCysIIIov  FPACysIIIov  RPDHap43ov | TCGATACCGTCGACCTACATACAGCTAAAAGTGTGTCGTAGTTTATTGG  ACTGGCAACAGTAGTGCCACCATCAGAAGAAGTATCCTTG  TCTTCTGATGGTGGCACTACTGTTGCCAGTCGAAGTACAAAATC  GGGAACAAAAGCTGGGTTTGTTTGTTTGTTTGTTTGAGATTTTTATAGTAA | Plasmid generation  CIp10-*HAP43_^481,503-634^* |
| FPUHap43ov  RPBCysIIIIov  FPACysIIIIov  RPDHap43ov | TCGATACCGTCGACCTACATACAGCTAAAAGTGTGTCGTAGTTTATTGG  CTTATAAGCATCTGCTGGAATGAAAATACCACTATTGCTCAG  GGTATTTTCATTCCAGCAGATGCTTATAAGACTTTGTCTCGTCAT  GGGAACAAAAGCTGGGTTTGTTTGTTTGTTTGTTTGAGATTTTTATAGTAA | Plasmid generation  CIp10-*HAP43_^583,585-634^* |
| URA-F2  RPF-1 | GGAGTTGGATTAGATGATAAAGGTGATGG  GAGCAGTGTACACACACACATCTTG | Conrormation of plasmid integration |
| RPF-2  M13R1 | CGCCAAAGAGTTTCCCCTATTATC  AGCGGATAACAATTTCACACAGGA | Conrormation of plasmid integration |
| Fhap  Rhap  4_R  5_R  6_R  7_R  8_F | TTGCAATAGCAGGATCACCA  TCGTAACACGTTGGCTACTGA  CAGTTTCAGTTTGGGGTTGAG  TCTTGGAAGCTGGCAATACA  GCTCAGAACCGACGAAGAGT  GCCATTTCCTTTATGTCCCTTT  AGGAGCCCACAAAAGACAAA | *HAP43* expression check from cDNA samples |
| EF1B-F  EF1B-R | AGTCATTGAACGAATTCTTGGCTG  TCTTCATCAACTTCATCATCAGAACC | gDNA contamination |
| ACT1-F  ACT1-R  rtFCCC1  rtRCCC1  rtFRBT5  rtRRBT5  rtFHAP43  rtRHAP43  rtFSIT1  rtRSIT1  rtFHMX1  rtRHMX1  rtFFRP1  rtRFRP1  rtFFRE9  rtRFRE9  rtFPGA7  rtRPGA7  rtFSEF1  rtRSEF1  rtFSFU1  rtRSFU1  rtFFTR1  rtRFTR1  rtFFTR2  rtRFTR2  rtFFTH1  rtRFTH1 | TCAGACCAGCTGATTTAGGTTTG  GTGAACAATGGATGGACCAG  TGGGTGAAGGTTGTCCAAAT  CAAGAACACCAATCCCCAAG  CTGCTGAAAGTTCTGCACCA  GCTTCAACGGAAACAGAAGC  TGAACAACCCTCACCAATCA  TCTTGGAAGCTGGCAATACA  TGCTATGTGGATGGTTGCAT  CAATGCCGATGAAATCACAG  AATTTGCCCTTGCTTTGAGA  GTCTTGCTCGGCTTTACCAG  GGGTGGTGCATTTACATTCC  TCCAGCAAACTGTTTCAACG  AAGAAGGCGATGGTACGAAA  CAATGCCGATGAAATCACAG  CAGGATGTTTCTGCGTGATG  GCCAAGAATCTGGCACTAGC  GTTGCTCCATTTGCATCTCA  CAAACCAACCCAAAAAGTCG  ATCAAACAGCACCGCTCTCT  GGTCCGGGTCCATTAGGTAT  GCCGGTATCGTTGTTGGTGC  GTGTTGGTTTCGAAATACCAAATACCTC  TGTGGTCTTGCAGTGGGTG  GAGGTGTCTGGTTCTTTGAGAGTTA  GCTGGTTTGTTCTCCAGAGG  ACCGTGACCAGTCTCACTCA | qRT-PCR |
